# Preoperative bi-fractionated accelerated radiation therapy for combined treatment of locally advanced rectal cancer in a consectutive series of unselected patients

**DOI:** 10.1186/1477-7800-4-23

**Published:** 2007-09-20

**Authors:** Roberto Biffi, Hugo Marsiglia, Barbara Jereczek Fossa, Maria Cristina Leonardi, Domenico Cante, Roberta Lazzari, Antonio Chiappa, Sabine Cenciarelli, Bruno Andreoni, Maria Giulia Zampino, Roberto Orecchia

**Affiliations:** 1 Dept. of General Surgery-European Institute of Oncology and University of Milano, Via Ripamonti, 435, Italy; 2 Dept. of Radiotherapy-European Institute of Oncology and University of Milano, Via Ripamonti, 435, Italy; 3 Dept. of Radiotherapy, Institute Gustav Roussy, Villejuif, France; 4 Dept. Of Oncolgy-European Institute of Oncology-Milano, Via Ripamonti, 435. Italy

## Abstract

**Background:**

although preoperative RT (Radiation Therapy) is becoming the preferred approach for combined treatment of locally advanced rectal adenocarcinoma, no regimen can be now considered as a standard. Since the toxicity of preoperative RT isn't yet completely known, and the advantages of preoperative RT could be counterbalanced by increased postoperative morbidity and mortality, a monocentre series of preoperative bifractionated accelerated RT was retrospectively reviewed to clarify toxicity and outcomes after a prolonged follow up.

**Methods:**

patients were screened following these eligibility criteria: histology-proven adenocarcinoma of the rectum; distal tumour extent at 12 cm or less from the anal verge; clinical stage T3–4/anyN, or anyT/N1–2; ECOG Performance Status 0–2. A total dose of 41.6 Gy (26 twice daily fractions of 1.6 Gy) was delivered. Surgery was carried out 17 ± 2 days after RT completion, adopting the total mesorectal excision technique.

**Results:**

24 men and 23 women were enrolled; median age was 55 years (r.: 39–77). Twenty-eight patients were stage II and 19 stage III. 9 patients suffered from a recurrent tumour. 2 patients experienced a severe grade 4 gastrointestinal toxicity (a colo-vaginal fistula and an intestinal obstruction, both successfully treated). Operative mortality was nil; postoperative early complications occurred in 13 cases; mean length of hospital stay was 15 days. After a mean follow up of 44 months (r.: 18–84) 8 patients had deceased for recurrent disease, 15 were alive with a disease progression (2 pelvic recurrences and 13 pure distant deposits) and 24 were alive, without disease. The 5-year actuarial overall survival was 74.2%, the disease-free survival 62.9% and the regional control rate 84.7%. Long-term complications included 1 case of radiation enteritis requiring surgery, 2 cases of anastomotic stricture and 3 cases of bladder incontinence.

**Conclusion:**

bifractionated accelerated RT administered in the preoperative setting to patients bearing locally advanced rectal cancer is reliable and safe, as its immediate and late toxicity (mainly infectious) is acceptably low and long-term survivals are achievable. These findings support the increasing use of preoperative RT for treatment of this malignancy in experienced centres. Ongoing multicentric trials are expected to address still unsolved issues, including the benefit of CT adjunct to preoperative RT.

## Background

Surgery remains the primary modality of treatment for rectal cancer and is the only therapy required for early-stage disease. Locally advanced rectal adenocarcinoma (i.e. resectable cancer with transmural spread and/or lymphatic nodes involvement) continues indeed to represent a major challenge to surgeons. Although remarkable progress has been made during the past two decades in improving surgical techniques, tumours in the lower half of the rectum pose specific technical problems, and a radical R0 resection of the tumour and its lymphatic component may be difficult to obtain, especially when a huge lesion is located in a very narrow pelvis of a male patient. Several studies have recently focused attention on the importance of standardized surgical technique of total mesorectal excision [1,2] as well as its integration with chemo-(CT) and radiation therapy (RT) for optimal treatment of this malignancy [3,4]. Although preoperative RT is becoming the preferred approach to locally advanced rectal cancer in many Institutions and large randomised trials have shown that preoperative RT can substantially decrease local failure rates and slightly improve overall survival [5,6], no RT regimen can be now considered as a standard, and ongoing studies are still addressing some major unresolved issues, including the optimal timing of RT (preop- vs postoperative) and the relative merits of short vs long-course RT. A major concern raised from a recent meta-analysis, showing a consistent increase in noncancer-related mortality for patients receiving preoperative RT [7]; since the toxicity of preoperative RT seems to be not yet completely known, and the advantages of preoperative RT could be counterbalanced by increased postoperative morbidity and mortality, as seen in some trials [8], we decided to retrospectively review our series of preoperative bi-fractionated accelerated RT in locally advanced rectal cancer, in the attempt to identify risk factors for early and late toxicity (if any), and to define most significant oncologic and functional outcomes after a prolonged follow up.

## Methods

After being informed and giving their consent, 47 consecutive patients from the European Institute of Oncology in Milan were enrolled in this study. The eligibility criteria included the following: histology-proven adenocarcinoma of the rectum; distal tumour extent within 12 cm from the anal verge; clinical stage T3–4/anyN, or anyT/N1–2 for primary or recurrent disease as well; no evidence of distant deposits; ECOG Performance Status 0–2; age >18 years; no prior or concurrent malignancy, and finally no prior RT. Adjuvant CT was allowed for eligible patients, suffering from recurrent carcinoma of the rectum.

Pre-treatment evaluation included complete physical examination, digital rectal examination, common laboratory biochemical test, CEA (Carcinoembryonic Antigen) determination, colonoscopy, CT-scan (Computerized Tomography) of the thorax, abdomen and pelvis, and EUS (endoscopic rectal ultrasonography). Total dose delivered was 41.6 Gy in 26 fractions of 1.6 Gy, two times daily with at least a 6-h interval. The target volume included the tumour and each enlarged lymph node (if any), with a margin of at least 3 cm, perirectal and internal iliac lymph nodes, the presacral area and surrounding organs considered to be involved by tumour. A four-field technique (box) was always used. Dosimetry was optimised by means of a treatment-planning computerized program, on the basis of either patient's contours or dosimetric CT scan. High energy photons of at least 15 MV were used.

Surgery was scheduled after a rest period of 17 ± 2 days from RT completion. Total mesorectal excision technique was always adopted. Adjuvant CT (5 FU + Folinic Acid bolus) for a 6-month period was planned for patients who had T4 or N+ at pathology report of surgical specimen.

## Results

Table 1 summarizes patients' population pertinent characteristics. 47 patients were treated (24 men and 23 women); the median age was 55 years (r.: 39–77). Pre-treatment clinical staging included 28 cases stage II and 19 stage III. 9 patients suffered from a recurrent tumour. Median distance between the lower tumour edge and the anal verge was 5 cm (range: < 1 – 12).

**Table 1:** patients' population pertinent characteristics

No. of patients treated	47
Male: Female ratio	24 : 23
Mean age (range)	55 yrs. (39–77)
Primary tumours : recurrences ratio	38 : 9
cTNM Stage II : stage III ratio	28 : 19
Mean distance between tumour edge and anal verge (range)	5.0 cm (<1 – 12)
No. of patients who completed RT programme as protocol	44 (93.6%)

Three patients did not complete the planned RT schedule as a consequence of early toxicity; in these cases the dose administered was 32, 40 and 40.8 Gy respectively.

Table 2 shows the observed toxicity; severe grade 4 gastrointestinal reactions included a colo-vaginal fistula, while 1 patient suffered from grade 3 toxicity (intestinal sub-obstruction). Both were successfully treated. 9 cases of grade 2 and 19 cases of grade 1 gastrointestinal reactions were detected. One case of grade 2 and 15 cases of grade 1 urogenital morbidity were observed. 6 cases of grade 2 and 6 cases of grade 1 haematological toxicity occurred. Finally, one grade 2 and 7 grade 1 skin toxicity were observed.

**Table 2:** observed acute toxicity from preoperative radiation therapy

**Type**	**Grade 1**	**Grade 2**	**Grade 3**	**Grade 4**
Gastrointestinal	19	9	1 (intestinal obstruction)*	1 (colovaginal fistula)**
Urogenital	15	1	-	-
Haematologic	6	6	-	-
Skin	7	1	-	-

* successful conservative treatment; ** successful en-bloc resection at the time of programmed surgery

Surgery was performed at a mean interval of 17 ± 2 days after RT completion, always adopting the total mesorectal excision technique. Table 3 lists the main surgical features; abdomino-perineal resection (APR) was carried out in 14 patients, and low anterior resection with mechanical anastomosis in 33. 16 patients had blood peroperative transfusions; a mean of 2 units of packed red blood cells were administered (r. = 0–5). All operated patients could benefit of a curative surgery, expressed by an R0 procedure (proximal, distal and circumferential margins free of disease; the circumferential free margin resulted less than 1 mm in 9 cases).

**Table 3:** main surgical and pathology features in 47 operated patients

**Parameter**	**No**.	**%**
Low anterior resection	33	70.2
Abdomino-perineal resection	14	29.8
En-bloc resection of other organs	5	10.6
R-0 operation Circumferential free margin < 1 mm	47 9	100 19.1
Circumferential free margin < 1 mm	9	19.1
Patients having blood peroperative transfusions (mean: 2 PRBC; r: 0–5)	9	19.1
Downstaging (ypT1-2 ypN0)	3	6.3
ypT1 – ypT2	8	17.0
ypT3 – ypT4	39	83.0
Mean length of hospital stay (days; r)	15 (8–35)	

None patient obtained a complete pathology response to the treatment. Downstaging, expressed as pathologic ypT1–2, ypN0 stage, occurred in 3 patients only. A post-RT pathology stage ypT1 was detected in 1 case, ypT2 in 7, ypT3 in 33 and ypT4 in 6. Positive nodes were detected in 33 patients. Mean length of hospital stay was 15 days (range: 8–35). YpT4-any N and N positive patients underwent adjuvant chemotherapy for a median of 6 months (5 FU + Folinic Acid bolus).

Postoperative complications occurred in 13 cases (a detailed list is presented in Table 4): two anastomotic leaks (both requiring a re-operation), two cases of prolonged postoperative ileus, one pelvic abscess (causative agent: *P. aeruginosa*) and eight cases of surgical site infection (SSI) grade I-II, according to CDC classification (perineal and/or laparotomy wound).

**Table 4:** postoperative early complications

**Type**	**No. of cases**	**%**
Anastomotic leak	2 (out of 33)	6.0
Prolonged postoperative ileus	2 (out of 47)	4.2
Abdominal abscess	1 (out of 47)	2.1
Surgical site infections (grade I-II)*	8 (out of 47)	17.0
All	13 (out of 47)	27.6

*according to CDC classification of Surgical Site Infections

After a mean follow up of 44 months (range: 18–84) (Table 5), six patients experienced severe late complications: three cases of grade 3 gastrointestinal toxicity (1 radiation enteritis requiring surgery and 2 cases of anastomotic stricture requiring serial endoscopic dilatations) and three cases of grade 3 urogenital toxicity (vescical incontinence). No case of severe cardiovascular toxicity occurred. 8 patients (6 of them affected from recurrence of previous rectal cancer) had deceased for recurrent disease (pelvic recurrence and concomitant distant metastases), 15 were alive with a disease progression (2 pelvic recurrences and 13 pure distant deposits) and 24 were alive, without disease. The 5 year actuarial overall survival was 74.2%, the disease-free survival 62.9% and the regional control rate 84.7%.

**Table 5:** long term results (mean follow up: 44 mo., r. = 18–84)

**Parameter**	**No**.	**%**
Deceased (progression of disease)	8	17.0
Primary tumour	2	25
Recurrent disease	6	75
Alive without disease	24	51.0
Alive with disease	15	31.9
Overall local recurrences	10	21.2
Pure distant metastases	13*	27.6
Urogenital toxicity – grade 3	3**	6.3
Gastrointestinal toxicity – grade 3	3***	6.3

* liver 7 cases, lung 4 cases, peritoneum and lymphnodes 2 cases

** bladder incontinence

*** 1 case of radiation enteritis requiring surgery, 2 cases of anastomotic stricture requiring serial endoscopic dilatations

## Discussion

At present, a variety of innovative RT schedules aiming at obtaining minimal treatment toxicity are being tested, including preoperative bi-fractionated accelerated RT. Theoretic common advantages of long- and short-course of preoperative RT include the better tumour radio sensitivity, coming from improved oxygenation of presurgical field, the reduction in tumour seeding by surgical handling and perhaps the reduced toxicity, due to the lesser amount of radiated small bowel. A recent meta-analysis of studies dealing with adjuvant RT for rectal cancer, including over 8,000 patients, has reported a reduction of local recurrence risk by almost 50% by preoperative treatment, independently from fractionation schedules, compared to 37% of risk reduction for postoperative RT [7]. Practical and economic considerations provided a major effort for developing short-course treatments, as the tumoricidal effect of short-course RT (25 Gy in one week) seems to be equal to that of long-course (42 to 50 Gy over four to six weeks), using a variety of schedules [9-11]. Unfortunately, a single randomised study has compared preoperative short-course with postoperative RT, delivered over 8 weeks [12]; the reduced local recurrence rate with the former does not mean a inherent superiority of short vs long-course and adds little to the debate regarding this issue. However, a major concern could be the price for short-course preoperative RT, in terms of increased short- and long-term toxicity, as previously observed with short-course RT at other sites. This concern has been highlighted in the above mentioned meta-analysis, which showed a significant increase in noncancer-related mortality at one year of follow up, mainly as a consequence of cardiovascular events in older patients [7]. We did not observe similar events in our experience, and is likely that could be the result of outdated techniques, as there was no increase in postoperative mortality in patients receiving preoperative RT according to modern techniques in either the Swedish Rectal Cancer Trial or the Dutch Trial. With respect to early toxicity, the investigators from the Swedish trial reported an increased frequency of postoperative fistulas and femoral neck and pelvic fractures; moreover, for patients who underwent a conservative surgery, a significantly altered residual sphincter function was found [5]. The Dutch group reported an increase in perineal complications (essentially infectious) in patients receiving preoperative RT compared to surgery alone controls [6]. Our own data confirm these findings: the vast majority of early observed toxicity is related to infectious complications of surgical wounds, whereas anastomotic leaks and pelvic abscess are less relevant.

Our single case of late small bowel toxicity confirms another previous evidence, coming from randomised trials and retrospective experiences, that preop-RT may have fewer adverse effects on long-term bowel function than postoperative therapy [13,14]. One possible explanation could be the fact that the radiated rectosgmoid is removed after neoadjuvant therapy; moreover, this could be the result of a lower radiation dosage to the small bowel, as it is much easier to exclude the small intestine from the radiation field in a pelvis free of surgical adhesions by proper positioning of the patients [15]. Moreover, postoperative treatment is associated with increased frequency of bowel movements per day, higher rate of incontinence and increased use of antidiarrhea medications [16]. In addition, a large German multi-institutional study reported that only 37% of expected patients were referred for postoperative adjuvant treatment, reflecting a surgeons' policy based on patients' individual risk factors evaluation instead of TNM stage [17]. In our non-randomised study, no patients who were continent before treatment developed incontinence, thus comparing favourably with the incontinence rates associated with postoperative adjuvant treatment.

A possible benefit of preoperative therapy is the potential for downstaging, thus increasing resectability rate and the possibility of sphincter-preserving surgery. Although the extent of downstaging appears related to the time interval between completion of preop RT and surgery, the two major studies of preoperative radiation therapy in rectal cancer (Swedish [5] and Dutch [6] trials) scheduled surgery after 1 week and 10 days respectively after the end of radiation treatment; moreover, in the Swedish trial more patients in the preoperative RT group had Dukes stage A or B cancers, whereas in our series only patients with locally advanced or recurrent disease have been treated. In the larger Dutch study no difference in the rates of positive circumferential margins has been detected (16% in the preoperative RT vs 19% of surgery alone); our own data confirm this evidence: a short course of preop-RT and a short time-interval before surgery are not able to produce a significant pathologic downstaging, and complete responses should not be expected. Our finding of 9 out 47 patients with minimal circumferential free margin at surgery (less than 1 mm) confirms that the biological effect of radiation can extend beyond the anatomic boundaries of the lesion, with minimal damage to surrounding normal tissues.

Minsky et al. studied 22 patients with distal rectal cancers that had been deemed to require APR for cure, and found that preoperative RT resulted in significant tumour downstaging, enabling approximately 90% of patients to undergo sphincter preservation surgery [18]. Similarly, Rouanet et al. used preoperative irradiation to treat 37 patients with rectal cancer that was thought to require an APR [19]; sphincter salvage was possible in 78% of patients. Francois and co-workers [20], in the only study specifically designed to explore this issue, randomised a total of 201 patients to either surgery after 2 weeks or after 6–8 weeks from the completion of the RT; both clinical and pathologic downstaging were significantly higher in the longer interval group.

Nonetheless, it's our opinion that achieving a microscopic tumour-free circumferential margin is an important factor affecting local pelvic recurrence, and the surgeon's ability to obtain tumour clearance can be challenged by the physical constraints of a narrow pelvis, particularly in male patients. A short course of preop-RT offers the advantage of decreasing the size ("downsizing") of the tumour before surgery, increasing the resectability rate of lesions which were previously deemed unresectable with curative intent or at high risk of R1–R2 procedures. In our series, patients with recurrent tumours and those with huge masses deeply invading the mesorectum had local recurrence rates that remain very high, in spite of preoperative RT administration; that's why our efforts are now directed to obtain a better local control of these huge lesions by means of combined preoperative CT-RT treatment, using higher doses and longer time interval, in agreement with other groups' reported experiences [21].

## Conclusion

In conclusion, bi-fractionated accelerated RT administered in the preoperative setting to patients suffering from locally advanced rectal cancer was safe in terms of early and long-term toxicity, the latter being expressed as acceptably low postoperative complications' rate (mainly infectious) and toxic effects on small bowel. These findings support the increasing use of preoperative RT in the treatment of locally advanced rectal carcinoma; ongoing trials are expected to address major unsolved issues, including the role of combined modality treatment (RT plus CT) in the preoperative setting.
